# Mediastinal pancreatic pseudocyst diagnosed based on black pleural effusion

**DOI:** 10.1002/rcr2.1195

**Published:** 2023-07-25

**Authors:** Isana Katayama, Arisa Komatsu, Takayasu Watanabe, Daisuke Hayakawa, Naoko Iwakami, Takuya Genda, Shin‐ichiro Iwakami, Kazuhisa Takahashi

**Affiliations:** ^1^ Department of Respiratory Medicine Juntendo University Graduate School of Medicine Tokyo Japan; ^2^ Department of Respiratory Medicine Juntendo Shizuoka Hospital Shizuoka Japan; ^3^ Department of Gastroenterology Juntendo Shizuoka Hospital Shizuoka Japan

**Keywords:** black pleural effusion, mediastinal pancreatic pseudocyst, pancreatic pleural fluid

## Abstract

Mediastinal pancreatic pseudocysts are rare complications of pancreatitis associated with alcohol consumption. Here, we report a case of mediastinal pancreatic pseudocyst. A 61‐year‐old Japanese woman presented to our hospital with epigastric pain and dyspnea. A chest radiograph revealed right‐sided massive pleural effusion. Thoracentesis retrieved black pleural fluid with remarkably high fluid amylase levels were. Thoracic computed tomography (CT) after drainage revealed encapsulated fluid. Magnetic resonance cholangiopancreatography (MRCP) and endoscopic retrograde cholangiopancreatography (ERCP) were performed because abdominal CT and ultrasonography did not reveal any pancreatic problems. MRCP showed cystic masses and pancreatic tail cysts extending to the stomach and lower oesophagus. ERCP confirmed leakage of contrast medium from the pancreatic tail into the retroperitoneum. We diagnosed the patient with a pancreatic pseudocyst extending to the mediastinum. A mediastinal pancreatic pseudocyst should be considered a differential diagnosis in patients with black pleural fluid with a high amylase level.

## Graphical abstract

Mediastinal pancreatic pseudocysts are rare complications of pancreatitis associated with alcohol consumption. Here, we report a case of mediastinal pancreatic pseudocyst in a patient who presented with black pleural fluid with a high amylase level.

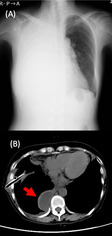

## INTRODUCTION

Pancreatic pseudocysts extending to the mediastinum are unusual complications of pancreatitis. Previous reports have shown that mediastinal pancreatic pseudocysts are associated with acute or chronic pancreatitis with heavy alcohol consumption. However, reports of mediastinal pancreatic pseudocysts with no history of alcohol consumption are rare. We report a case of mediastinal pancreatic pseudocyst diagnosed by focusing on two important characteristics of the pleural fluid.

## CASE REPORT

**FIGURE 1:**
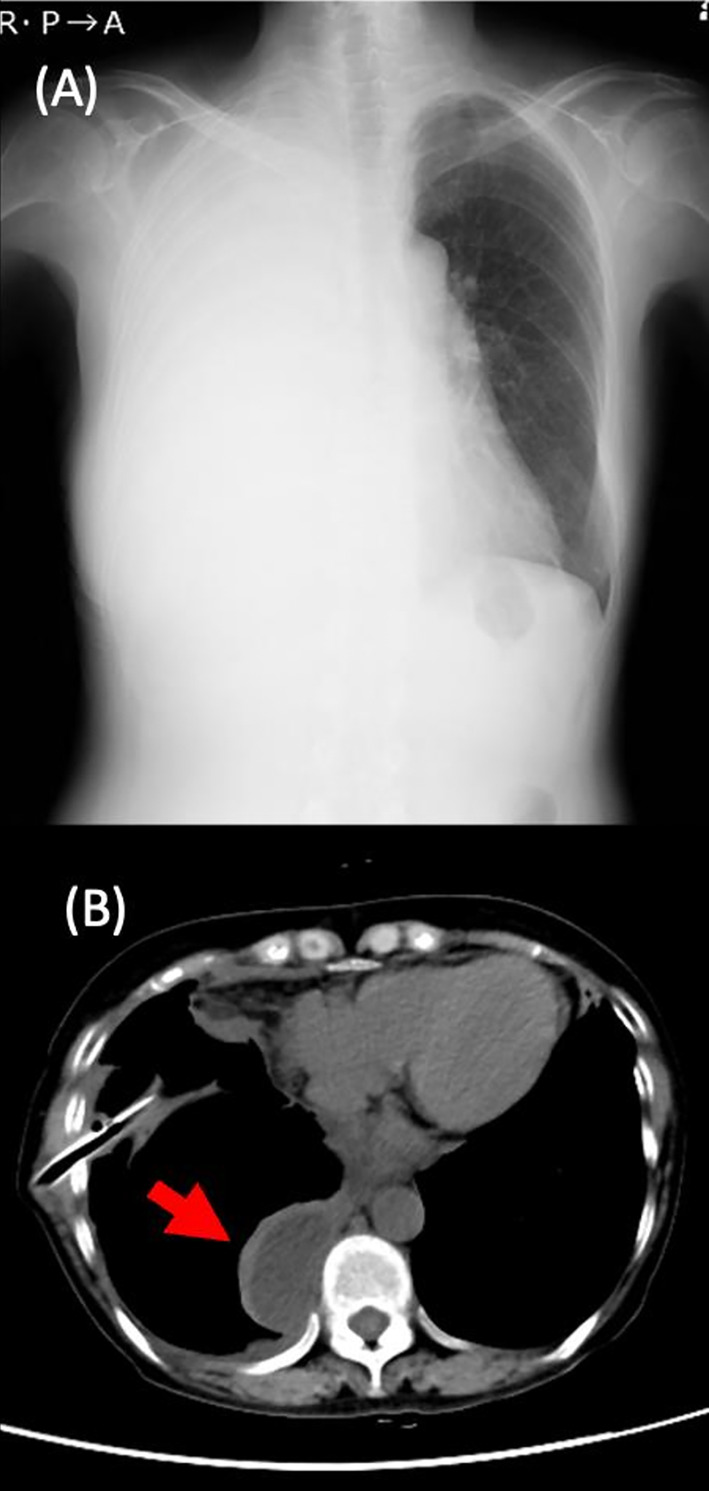
(A) Chest radiograph obtained on the day of admission showing massive right‐sided pleural effusion (23 Dec 20XX‐1). (B) Thoracic computed tomography after chest tube drainage shows an encapsulated pleural effusion (Red arrow) (4 Jan 20XX).

**FIGURE 2:**
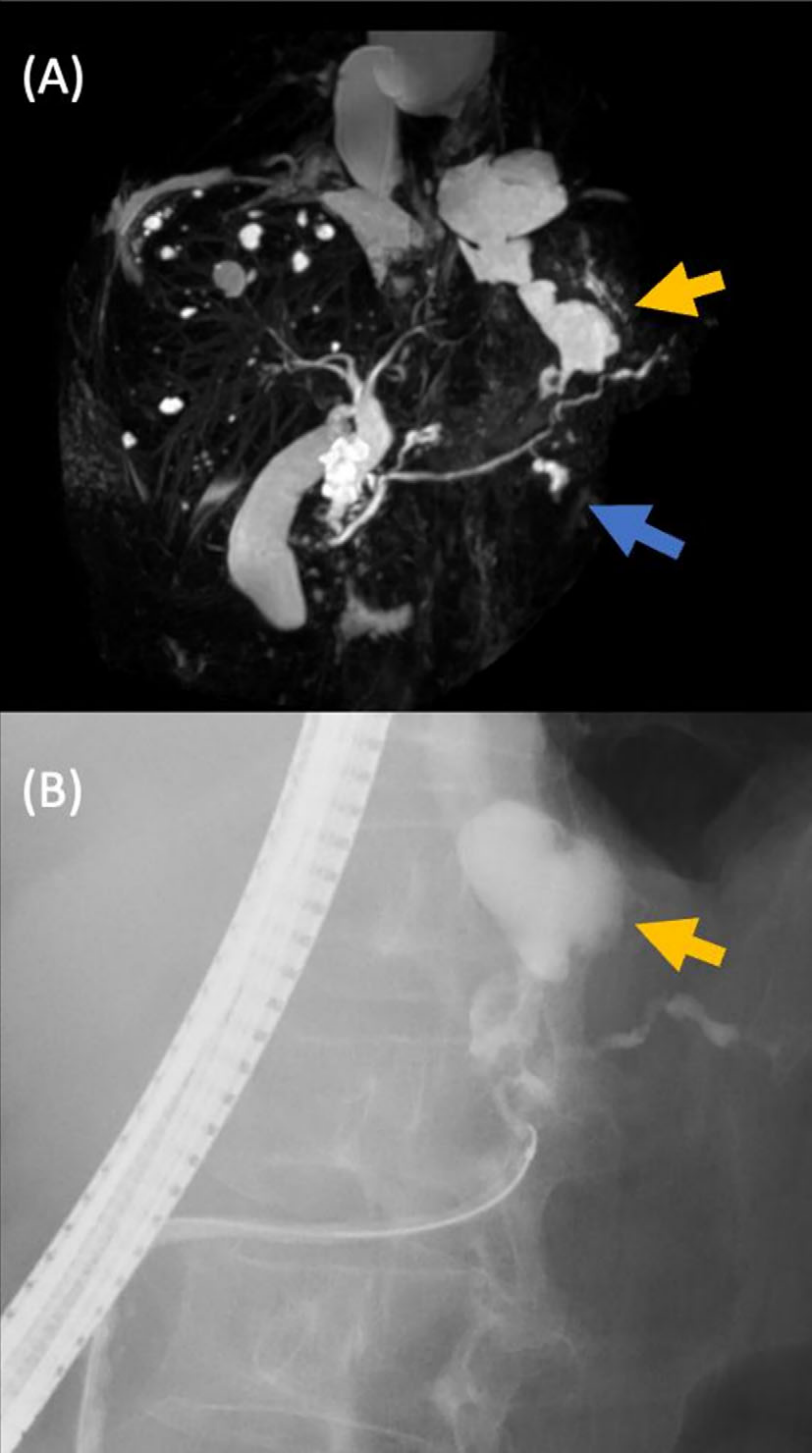
(A) MRCP shows cystic masses (Blue arrow) and pancreatic tail cysts extending to the stomach and lower oesophagus (Yellow arrow) (7 Jan 20XX). (B) ERCP shows leakage of the contrast medium from the pancreatic tail into the retroperitoneum (Yellow arrow) (14 Jan 20XX).

A 61‐year‐old woman developed gradual‐onset epigastric pain and exertional dyspnea. She presented to our hospital 1 month after the onset of symptoms. The patient did not consume alcohol. Her medical and family history was insignificant, and she did not take any medication or nutritional supplements. On examination, she was in distress, and O_2_ saturation was 92% on room air. Her blood pressure, pulse and body temperature were 124/71 mmHg, 78/min and 36.9°C, respectively. Chest examination revealed dullness on percussion and decreased breath sounds in the right lung. The epigastric pain radiated to the back, with no rebound or guarding. The main results of a blood analysis were as follows: white blood cell count 7000/μL, haemoglobin 12.2 g/dL, total protein 6.4 g/dL, albumin 2.5 g/dL, lactate dehydrogenase 199 IU/L, amylase 1288 IU/L (100% pancreatic isotype), C‐reactive protein 19.4 mg/dL, and CA19‐9104 ng/mL. Chest radiography revealed a large, right‐sided pleural effusion (Figure 1A). Thoracic computed tomography (CT) revealed massive pleural effusion in the right lung. Thoracentesis was performed, and the aspirated pleural fluid was black and exudative. Pleural fluid analysis was as follows: pH 7.35, total protein 3.9 g/dL, lactate dehydrogenase 546 IU/L, glucose 101 mg/dL, albumin 1.8 g/dL, amylase 55,128 IU/L and CA19‐9586 ng/mL. The cytological examination was negative for malignant cells, and culture of the pleural fluid did not reveal any significant pathogens. Chest tube drainage was performed for relief. The patient's symptoms gradually improved. Thoracic and abdominal CT after drainage showed a small amount of encapsulated pleural fluid (Figure 1B), no lung masses, and a normal pancreas. However, we considered that the pleural fluid originated from the pancreas since serum and pleural amylase levels were highly elevated and the pleural fluid was black. Magnetic resonance cholangiopancreatography (MRCP) revealed cystic masses near the main pancreatic duct and cysts of the pancreatic tail extending consecutively to the stomach and lower oesophagus (Figure 2A). We suspected that the pancreatic pseudocysts traversed the retroperitoneum and oesophageal hiatus to the right thoracic cavity via the pleura. Endoscopic retrograde cholangiopancreatography (ERCP) confirmed leakage of the contrast medium from the pancreatic tail into the retroperitoneum (Figure 2B); however, no leakage of the contrast medium into the thoracic cavity was observed. Since ERCP showed leakage of contrast medium, MRCP showed a pancreatic pseudocyst contiguous to the lower oesophagus, and pleural fluid amylase was elevated, suggesting a pancreatic origin, we concluded that the pancreatic pseudocysts had perforated the mediastinum. Endoscopic pancreatic stent placement to treat the mediastinal pancreatic pseudocysts was unsuccessful. A somatostatin analogue was then administered for 22 days to suppress pancreatic exocrine secretion. The patient's symptoms and pleural fluid volume improved gradually.

## DISCUSSION

Pancreatic pseudocysts are a complication of acute or chronic pancreatitis. Mediastinal pancreatic pseudocysts are rare. The incidence of pancreaticopleural fistulas in cases of pancreatitis is estimated to range from 0.4% to 4.5%.^1^ In our case, the patient had an insignificant medical history of pancreatitis and other diseases and no history of alcohol consumption. Abdominal CT and ultrasonography performed on admission revealed no evidence of pancreatitis.

However, we concluded that the pleural fluid originated from the pancreas by focusing on two characteristics of the pleural fluid.

First, the pleural fluid was black in colour. Pancreatic pleural fluid is often black, which is thought to have caused damage to the pleura by the pancreatic fluid, resulting in haemorrhage and haemolysis. Pancreatic fluid, fungal infection (*Aspergillus niger* and *Rhizopus oryzae*) and malignant melanoma should be considered as differential diagnoses in case of black pleural effusion.^2^

Second, the pleural fluid amylase level was remarkably high (55,128 IU/L). Tay reported that the mean amylase level in the pleural fluid of patients with pancreaticopleural fistulas may exceed 10,000 IU/L.^3^ Pancreatic fluid may enter the mediastinum via the aortic or oesophageal hiatus. In our case, pancreatic fluid was suspected to have passed through the oesophageal hiatus.

We thought these two pleural findings were important clues that the pleural fluid originated from the pancreas. Abdominal CT and ultrasonography did not reveal any abnormal pancreatic findings. Magnetic resonance imaging is useful for demonstrating the fistulous tract.^4^ In this case, MRCP showed cystic masses near the main pancreatic duct, and cysts of the pancreatic tail extended to the stomach and lower oesophagus. If ERCP confirms leakage of contrast medium into the thoracic cavity, a definitive diagnosis of perforation of pancreatic pseudocysts into the mediastinum can be made, but this is not always easy.

Mediastinal pancreatic pseudocysts are treated with surgical resection or endoscopic intervention, such as pancreatic stent placement, and somatostatin analogues have been reported to be efficacious for the treatment of pancreatic pseudocysts that are refractory to conservative therapies. Somatostatin is a peptide hormone that inhibits the release of insulin, glucagon, gastrin, thyroid‐ stimulating hormone, adrenocorticotropic hormone, secretin, cholecystokinin, pepsin and renin.^5^

If a patient has a black pleural effusion, amylase level of the pleural fluid should be measured, and pleural effusion due to mediastinal pancreatic pseudocyst should be considered as a differential diagnosis.

## AUTHOR CONTRIBUTIONS

Isana Katayama was the treating physician and first author of this article. Arisa Komatsu, Takayasu Watanabe, Daisuke Hayakawa, and Naoko Iwakami discussed diagnostic methods and treatment strategies. Takuya Genda, Shin‐ichiro Iwakami, and Kazuhisa Takahashi supervised clinical practice and revised the manuscript.

## CONFLICT OF INTEREST STATEMENT

None declared.

## ETHICS STATEMENT

The authors declare that appropriate written informed consent was obtained for the publication of this manuscript and accompanying images. This report was approved by the Ethics Committee of Juntendo Shizuoka Hospital.

## Data Availability

The data that support the findings of this study are available on request from the corresponding author. The data are not publicly available due to privacy or ethical restrictions.
